# Efficacy of a proactive health and safety risk management system in the fire service

**DOI:** 10.1186/s40621-018-0148-9

**Published:** 2018-04-16

**Authors:** Gerald S. Poplin, Stephanie Griffin, Keshia Pollack Porter, Joshua Mallett, Chengcheng Hu, Virginia Day-Nash, Jefferey L. Burgess

**Affiliations:** 1 Center for Applied Biomechanics, School of Engineering and Applied Sciences, University of Virginia, Charlottesville, USA; 2 Mel and Enid Zuckerman College of Public Health, University of Arizona, Tucson, USA; 3 The Johns Hopkins Bloomberg School of Public Health, Baltimore, USA

**Keywords:** Injury, Risk management, Firefighting, Workers’ compensation

## Abstract

**Background:**

This study evaluated the efficacy of a fire department proactive risk management program aimed at reducing firefighter injuries and their associated costs.

**Methods:**

Injury data were collected for the intervention fire department and a contemporary control department. Workers’ compensation claim frequency and costs were analyzed for the intervention fire department only. Total, exercise, patient transport, and fireground operations injury rates were calculated for both fire departments.

**Results:**

There was a post-intervention average annual reduction in injuries (13%), workers’ compensation injury claims (30%) and claims costs (21%). Median monthly injury rates comparing the post-intervention to the pre-intervention period did not show statistically significant changes in either the intervention or control fire department.

**Conclusions:**

Reduced workers’ compensation claims and costs were observed following the risk management intervention, but changes in injury rates were not statistically significant.

## Background

Many fire departments provide both firefighting and emergency medical services (EMS). In the United States (U.S.), these activities were associated with 91 fatalities in 2014 alone, and over 70,450 injuries annually from 2010 to 2012 (U.S. Fire Administration 2014; U.S. Fire Administration, 2015). Furthermore, annual U.S. fire service injury and injury prevention costs have been estimated at $2.8 to $7.8 billion (TriData Corporation, 2005). The most frequent activities associated with injury vary by department, including exercise, patient transport, fireground operations and training (Poplin et al., 2012; Burgess et al., 2014), supporting the need for an injury prevention approach that allows each department to prioritize the hazards confronted.

Proactive risk management (RM) is required in many parts of the world, including the European Union and Australia (Burgess et al., 2014; Poplin et al., 2008). However, only limited outcome data are available on implementation of risk-based health and safety systems, much of it in the mining industry. Specific to the (bituminous) coal mining industry, lost time injury incident rates were found to have decreased significantly (52–78%) over an 8-year period (1996–2003) following a change in the Australian regulatory structure from mainly compliance to RM-based, compared to a 20% decline in the U.S., which operates predominantly under compliance-based regulations (Poplin et al., 2008).

Specific to the fire service, RM has been enforced by regulation in the United Kingdom (U.K.) since the 1990’s. In an international comparison, a U.K. fire department was found to have lost-time injury rates for fireground operations and training markedly lower than the U.S., Australian, and Canadian fire departments (Burgess et al., 2014). There is no information on the effectiveness of RM in the U.S. fire service, where such an approach is voluntary. In 2009, researchers from the University of Arizona and Johns Hopkins University partnered with the Tucson Fire Department (FD) to introduce and apply a RM approach for workforce health and safety. The objective of this analysis is to evaluate the effect of this RM process on FD injuries and their associated costs.

## Methods

The study design and methods of the RM process applied to the FD has been previously described (Poplin et al., 2015) and were approved by the University of Arizona Institutional Review Board. In brief, a three-phased process involving hazard scoping, risk assessment, and implementation of prevention controls was systematically employed over approximately three years, followed by a one year observation period. The intervention began in February of 2010 with scoping and risk assessment phases conducted through the remainder of the initial calendar year (approximately 11 months). Three teams, each involving a full cross-section of commissioned field and administration personnel, were formed to assess the hazards and injuries related to physical exercise (PE), patient transport (PT) and fireground (FG) activities and operations, as they accounted for 32.9%, 16.9% and 10.2% of all FD injuries, respectively, over the six-year pre-intervention period 2004–2009 (Poplin et al., 2012). FG activities are comprised of tactical operations with respect to active fire suppression, including but not limited to rescue, ventilation, utility mitigation, salvage and overhaul. Both FG and PT responses relate to the activities performed from the time of emergency dispatch, to post-response clean-up and returning to an “in-service” status. At the conclusion of the risk assessment phase, 8 of 45 potential controls described elsewhere (Poplin et al., 2015) were selected for development and implementation (Table 1). A ninth potential control, peer safety check before post-suppression activities, was reviewed but not initiated as FD decided pre-existing controls could be better enforced. Controls were installed beginning January of 2011 and over the subsequent 24 months, based in part, on the availability of resources needed to approve, develop, apply and evaluate each strategy.

**Table 1:** Applied intervention^a^ control strategies and their relative timing

Selected control strategy	Time of Implementation (months)^b^
Physical Exercise (PE)	
Improve station exercise equipment and facilities	1
Increase role of Peer Fitness Trainers	4
Update and revise exercise standard operating procedure (SOP)	14
Patient Transport (PT)	
Test patient transfer devices	3
Establish chest compression rotation procedure during CPR	15
Create PT module for probationary firefighters	24
Fireground Operations (FG)	
Improve rehab protocols & adherence	4
Visual reminders for health and safety	4,19

^a^The intervention is the risk management application, which began in 2010

^b^Relative to the beginning of the control implementation phase in January, 2011

### Implemented physical exercise (PE) controls

Each fire station was equipped with updated exercise equipment, and equipment not meeting department safety requirements (e.g., no metal weightlifting plates on concrete floor and no personal equipment from home) was removed. Space permitting, most stations received new exercise equipment to meet a general standard of two cardio-machines (e.g., treadmill, rowing ergometer, stationary bike), strength equipment (e.g., benches, dumbbells, etc.), and functional movement equipment (e.g., multi-function cable gym). In addition, monthly maintenance forms for each fire station were updated to include monthly assessment of the exercise equipment and to alert the health and safety officers to any potential problems.

The role of Peer Fitness Trainers (PFTs) was also expanded to assist recruits and probationary officers, as well as commissioned individuals with identified needs, to exercise safely as previously described (Griffin et al., 2016). Daily workout routines during fire academy were restructured, and each probationary firefighter was assigned a PFT to work with him or her to promote appropriate exercise. Improvements to PFT involvement, coordination and certifications were also made to better prepare the PFTs to act as a resource for all FD commissioned employees. Prior to these structured improvement, the PFTs primary role was to assist in conducting and reporting a part of the annual fitness exam that required a submaximal treadmill test. A PFT could also be assigned to a firefighter in instances when the firefighter failed consecutive fitness test, requiring more personal guidance.

Despite the existence of a standard operating procedure (SOP) describing the requirements for physical fitness, the level of awareness, enforcement and adherence to the SOPs had previously been inconsistent throughout the FD. Therefore, a priority control was to revise and update the SOPs directed at on-duty PE and fitness. Although each workday continued to include a mandatory workout period of 60 to 90 min, the previous set workout times at the beginning of the shift were replaced by permitting the timing of each exercise session to be left to the discretion of the shift captain and consensus of the crew. The change provides latitude for the individual captain to structure the workday most efficiently for the shift’s obligations and crew’s working capacity, which vary on a daily basis. The focus of PE was also changed from individuals choosing their own exercise to a new model where employees completed some form of exercise that maintained one or more of the areas relevant to being “fit for duty.” A basic skeleton-structure for daily exercise routines was emphasized to include functional warm-up and mobility exercises (as opposed to static stretching), cardiovascular and focused conditioning exercises, and a cool-down period dedicated to recovery, flexibility and range of motion. Conditioning exercises (e.g., interval training, back squats, stair climbs) were targeted to emphasize muscular strength, flexibility, cardiovascular endurance, muscular endurance, core strength, balance and coordination.

### Implemented patient transport (PT) controls

A slide board and a carry strap for patient lift assist (Weiler et al., 2013) were outfitted on every FD gurney to improve access and help reduce lifting loads and risk of strain during the lateral transfer of a patient. In addition, although not one of the initial controls selected due to costs, the FD outfitted each ambulance with new electronic lift assist gurneys to reduce the strain and repetition of vertical loads in early 2013, too close to the end of the overall project intervention period to adequately measure its effect on injury rates.

Given that approximately 80% of FD responses entail some form of medical assessment, frequently followed by transport, the PT learning module for probationary firefighters was updated to reflect current equipment and the timing changed from halfway through the probationary year to immediately following the recruit academy. The curriculum included an emphasis on gurney design, proper operation and positioning during patient movement, and techniques for loading the gurney onto and off of the ambulance. Given the frequency of lifting and moving (equipment, patients, and obstacles) from various (and often not ideal) ergonomic positions, the need for improved and maintained fitness levels emphasizing core strength was also stressed.

Cardiopulmonary resuscitation (CPR) was also identified as frequently being a fatiguing activity, often performed in awkward and prolonged static positions, increasing the likelihood for injury for the responder and ineffective compressions to the patient. During CPR vital signs and patient assessment occurs approximately every two minutes, or 200 compressions. The SOP was updated to include rotating CPR responsibility every 200 compressions when appropriate personnel are available and prepared.

### Implemented Fireground (FG) controls

The “demobilization and clean-up” stage was identified as posing the greatest perceived risk for FG injury, based on both likelihood and consequence. The top FD priority was to emphasize and enforce existing protocols for the use of personal protective equipment (PPE), including empowering the safety officer to remove a firefighter from scene if they are not wearing appropriate PPE. Specifically, the re-donning of turnout gear, helmet and gloves was emphasized during post-suppression activities, when individual situational awareness may be depressed. Previously, adherence to the SOP for releasing employees back to work, which entailed meeting vital sign levels during rehabilitation (rehab), was inconsistent. Implemented interventions included further defining and enforcing of the rehab SOP, including empowering the rehab paramedic, and positioning the rehab “tent” (or ambulance) further away from the on-scene activities so that firefighters undergoing rehab are less likely to be mixed-in with the commotion of tactical operations. The changes in SOP also included the addition of a second medic truck (or additional paramedics) in the rehab area for larger fires (i.e., a multi-alarm fire response). Active cooling using forearm immersion in cold water for 15 min was recommended for heat-stressed firefighters (Burgess et al., 2012).

Visual reminders (e.g., posters, placards, and signage) were developed to reinforce awareness of some of the identified FG risks, and to help improve adherence to SOPs. A simple image and brief message (i.e., “Save your joints, use 3-points”) was placed on the inside of all apparatus doors to use three points of contact when entering or exiting the vehicle. As part of the post-suppression demobilization and clean-up, standard PPE signage (e.g., eyewear, gloves, face shield) and reflective tape was placed around hose towers and chemical cabinets. At a later time, a “hydration” awareness and information chart (i.e., poster) was placed in all bathrooms to lower the potential for dehydration, which could potentially affect all responses and activities of the individual.

### Comparison fire department

The fire department selected as a non-intervention contemporary reference (control) group had nearly 2.5 times the annual employee population as our intervention FD, but shared comparable demographic characteristics and distribution of emergency response types. The control fire department also had an SOP mandating exercise while on duty, similar to the intervention FD. Details on injury, personnel (number of commissioned employees, gender, ethnicity, etc.) and dispatching (types and frequency of emergency responses) were only available for the control fire department starting in 2008, while data for the intervention FD were available starting in 2004.

### Evaluating efficacy

Several metrics were assessed in order to better evaluate the overall utility of the RM approach. Specifically, efficacy of the intervention was measured by: (1) comparing department-level costs associated with injury; (2) comparing injury frequency and rates over time and between the FD and the control fire department; and (3) conducting a formal process evaluation for the RM methodology, itself. As part of the RM method, continual or regular risk assessment is necessary to ensure that no new or unanticipated adverse risks are observed with the introduction of the individual control measure. This assurance was conducted through periodic in-person observations from researchers, in addition to three surveys conducted during the implementation phase that assessed changes in awareness of controls, utility and general perceptions on safety. The qualitative process evaluation data have been published elsewhere (Poplin et al., 2015).

As previously described (Poplin et al. 2012), injuries consisted of both OSHA recordables, as well as injuries without immediate loss of function, which are documented in the event that an individual’s condition worsens. For the intervention FD only, workers’ compensation claims data were available for calendar years 2004–2013. A total of 1455 claims were provided by the FD’s third-party payer, of which 117 were deleted for not meeting the definition of injury used in this study. These included claims for such incidents as allergic reaction, exposures, faint/dizzy, illness, heat exhaustion, heart attack, and stress. Claims for civilian or non-commissioned personnel (*n* = 66) were also excluded. The workers’ compensation database included information about the claim type (indemnity or medical-only), claim status (open or closed), and the costs paid to date, reserve costs, and total costs. Indemnity claims include payment for lost time; medical only claims include costs for medical treatment. Open claims typically have a reserve cost associated with them to cover any future incurred costs. Total costs are the sum of paid and reserve costs. Descriptive statistics for the 1272 claims filed during the study period were calculated after adjusting costs to constant end-of-year 2013 dollars using the Bureau of Labor Statistics Consumer Price Index (U.S. BLS, 2014). Pre- and post-intervention costs were also discounted at rates of 3% and 7%, consistent with the recommendations of the Panel on Cost-Effectiveness in Health and Medicine to account for the time preference of money (Gold et al., 1996).

A statistical analysis of injury trends was conducted using a likelihood-based negative binomial time-series method for count data where the rate of injury can depend on past rates, past observations and covariate values. The intervention effect was modeled as a binary indicator (starting in February 2010) comparing pre- and post-intervention rates. An annual seasonal effect was adjusted for. For injuries of all types and that related to exercise, the total number of firefighters for each year, on the log scale, was considered as an exposure variable. For the injury count of the specific tasks of FG and PT, the log number of calls in each month, prorated based on the annual count, was evaluated in the model as an exposure variable. Injury and response data were not available for the control FD prior to 2008, thus time trends and intervention effect comparisons between the intervention and control FD were restricted to the time period from 2008 to 2013. A Wald test was used to test for intervention effect within each FD and also to compare the same effects between the two FDs. All tests were two-sided at the significance level of 0.05. The statistical software environment R and the R package ‘tscount’ were used to fit the time series models (Liboschik et al., 2017).

## Results

Annual FD personnel, injuries, and workers’ compensation claims and costs are listed in Table 2. Comparing the average of pre-intervention years (2004–2009) and post-intervention years (2010–2013), there was a 3% decline in FD personnel, a 13% decline in all reported injuries, a 30% decline in claims frequency, and a 21% decrease in mean workers’ compensation claims costs.

**Table 2:** FD workers’ compensation claims and costs (total incurred), 2004–2013

	Frequency count			Total Incurred, $US			
Year	Personnel	Injuries	Claims	Mean (SD)	Median	Max	Total
2004	530	126	123	4009 (12,138)	779	122,964	493,048
2005	577	126	117	2986 (8786)	438	70,343	349,082
2006	625	148	153	5414 (29,792)	787	358,468	828,281
2007	659	174	199	3929 (10,059)	720	93,576	781,865
2008	694	199	148	2543 (7191)	724	55,174	376,368
2009	667	124	128	3532 (9552)	545	68,103	452,041
Pre-intervention average	625	150	145	3780 (15.256)	664	358,468	546,831
2010	654	143	115	2393 (5825)	406	39,768	275,138
2011	605	140	111	7078 (17,708)	971	108,181	785,636
2012	589	110	86	2941 (8168)	549	47,408	252,888
2013	590	128	92	4496 (12,680)	741	101,956	413,607
Post-intervention average	610	130	101	4275 (12,212)	642	108,181	431,817
All years	6190	1418	1272	3937 (14,356)	661	358,468	5,007,954

The mean claims cost for all claims from 2004 to 2013 was $3937 (SD =14,356) with a range of $0–$358,468 and median of $661. In the six years prior to implementation of the RM program, the average annual total incurred injury claims costs were $546,781, compared to $431,817 in the four years following implementation. The 21% reduction in total incurred claims costs is similar to what was observed in medical (22% reduction) and indemnity (20% reduction) components of claims costs (Table 3). Discounting at 3% and 7% reduced the cost savings for total incurred claims cost to 19% and 16%, respectively, with similar results for medical and indemnity costs.

**Table 3:** Summary of changes, pre- and post-RM intervention

Intervention time period^a^	Commissioned Personnel (n)	Injuries (n)	Claims (n)	Average Total Incurred ($) (95% CI)	Average Medical ($) (95% CI)	Average Indemnity ($) (95% CI)
No Discounting						
Pre (mean)	625	150	145	$546,781 (329,421, 764,140)	$457,152 (263,469, 650,835)	$80,166 (41,697, 118,635)
Post (mean)	610	130	101	$431,817 (39,800, 823,834)	$357,498 (37,907, 677,089)	$64,204 (321, 128,087)
Decrease (%)	3	13	30	21	22	20
Discounted – 3%						
Pre (mean)				$508,758 (304,005, 713,510)	$425,062 (243,023, 607,102)	$75,018 (37,674, 112,363)
Post (mean)				$413,693 (31,560, 795,825)	$342,567 (30,988, 654,145)	$61,534 (− 939, 124,007)
Decrease (%)				19	19	18
Discounted – 7%						
Pre (mean)				$465,068 (272,218, 657,919)	$388,162 (217,890, 558,434)	$61,109 (32,567, 105,650)
Post (mean)				$391,971 (21,040, 762,903)	$324,676 (22,102, 627,251)	$58,323 (− 2445, 119,090)
Decrease (%)				16	16	16

^a^pre-intervention = 2004–2009, post-intervention = 2010–2013

**Fig. 1:**
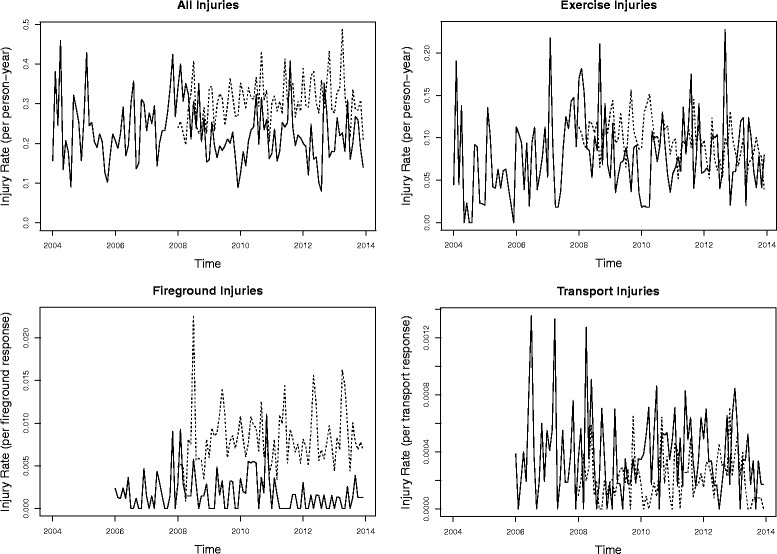
Injury rates, per person-month for both intervention (solid line) and control (dashed line) fire departments, 2004–2013

Median monthly injury rates for the intervention FD and control fire department are shown in Fig. 1. Injury data were available starting in 2004 for our intervention FD but only since 2008 for our control fire department. The implementation of RM formally started in 2010 for the FD, illustrated as a vertical line in each graph, however discussions regarding RM started before this date and the initiation of intervention components occurred at various intervals after this time (see Pollack et al. 2017 for additional details).

Using the described time-series methods to evaluate for potential intervention effects between 2010 and 2013 and 2004–2009 in our intervention FD, we observed a non-statistically significant decline of 8.1% (95% CI: -20.5, 6.3; *p* = 0.25) in the median monthly injury rate after the intervention began (Table 4). Non-statistically significant declines in PE (− 2.9%, 95% CI -25.4, 26.4) and FG (− 9.1%, 95% CI: -57.8, 95.8) injuries were also observed, and a non-significant increase in PT injuries (21.4%, 95% CI: -22.1, 89.4).

**Table 4:** Injury rates in intervention fire department 2004–2009 and 2010–2013, and change in median monthly injury rate between the two time periods

Task	Intervention FD			
	Pre-intervention (2004–2009)	Post-intervention (2010–2013)	Percent Change (95% CI)	*p*- value
Total (injury per person-year)	0.23900	0.21300	−8.1 (−20.5, 6.3)	0.25
Fireground^a^ (injury per response)	0.00187	0.00162	−9.1 (−57.8, 95.8)	0.81
Patient Transport^a^ (injury per response)	0.00037	0.00039	21.4 (−22.1, 89.4)	0.39
Exercise (injury per person-year)	0.07900	0.07800	−2.9 (− 25.5, 26.4)	0.83

^a^Number of response calls unavailable for 2004–2005, so pre-intervention consists of 2006–2009

Injury data were only available beginning in year 2008 for our control fire department. The median monthly injury rates comparing 2010–2013 to 2008–2009 are shown in Table 5. There was a non-statistically significant decline in the PE injury rate, while all injury rate as well as FG and PT rates increased over the same time frame. There was no statistically significant difference comparing the changes between the two fire departments.

**Table 5:** Injury rates in control fire department 2008–2009 and 2010–2013, and change in median monthly injury rate compared between the two time periods

Task	Control fire department			
	Pre-intervention (2008–2009)	Post-intervention (2010–2013)	Percent Change (95% CI)	*p*-value
Total (injury per person-year)	0.28200	0.31900	7.8 (− 5.6, 23.2)	0.27
Fireground (injury per response)	0.00790	0.00830	36.3 (7.6, 72.6)	0.01
Patient Transport (injury per response)	0.00021	0.00022	7.8 (−37.9, 87.0)	0.79
Exercise (injury per person-year)	0.10700	0.09200	−14.1 (−28.8, 3.8)	0.12

## Discussion

The current study aimed to assess the efficacy of introducing RM as a method for improving work health and safety in a career-based, U.S. metropolitan fire department. The RM process identified several control strategies that were targeted for implementation and the findings demonstrate decreased claims frequency and costs as well as a non-statistically significant reduction in overall injuries concurrent with the systematic application of the RM approach. The RM controls were put in place without substantially increased municipal allocation of funds to the department, due primarily to the economic recession that coincided with this time period. As previously reported, the RM program was also well received by the FD personnel (Poplin et al., 2015).

The results of the current study are consistent, albeit lesser in magnitude, than the findings of our previous international comparison of fire service injury rates, where a U.K. fire department with advanced RM was found to have lost-time injury rates during FG operations 4 to 60 times lower than U.S., Australian, and Canadian fire departments, as well as lower training injury rates (Burgess et al., 2014). However, proactive RM has been required by law in the U.K. since the 1970s and widely practiced by their fire service since the 1990s, entailing substantial allocation of resources and focus on a broader set of risks than those addressed in our study. The U.K. department also employed a civilian risk manager paired with a uniformed officer, and had front line uniformed fire service personnel trained and experienced in performing at least one risk assessment, none of which were implemented in the intervention FD.

Economic evaluation of health, safety and injury prevention interventions is uncommon in the fire service but the existing evidence indicates that departments may experience cost savings following implementation of health and wellness programs (Griffin et al., 2016; IAFF, 2008; Leffer and Grizzell, 2010; Kuehl et al., 2013). The International Association of Fire Fighters’ and the International Association of Fire Chiefs’ Wellness-Fitness Initiative (WFI) included an economic evaluation of workers’ compensation claims, days lost from work, costs per claim and total incurred costs comparing four WFI-participating departments and four non-participating departments (IAFF, 2008). Over the fourteen year study period, participating departments experienced a 5% increase in average claims costs and a 3% increase in total incurred costs compared to the 22% increase in average claims costs and 58% increase in total incurred costs experienced by non-participating departments (IAFF, 2008). Leffer and Grizzell (2010) reported a 60% reduction in injuries, a reduction in the number of obese firefighters, and a cost savings of $4.60 for every dollar invested over two years post-implementation of a physician organized wellness regime (POWR), based on the cost savings of avoided injury and lost time. An economic evaluation of the Promoting Healthy Living: Assessing More Effects (PHLAME) intervention revealed a significant reduction in workers’ compensation claims and medical costs among two participating departments compared to two control departments, yielding a beneficial return on investment of $4.61 for the team-based intervention (Kuehl et al., 2013). The potential benefits of a PFT intervention was highlighted in our recent study that showed recruit and probationary firefighters experienced significantly fewer injuries and filed fewer claims, resulting in a cost savings of nearly $33,000 from avoided injury and reduced claims costs over the 17-month study period (Griffin et al., 2016).

Our study measured a non-statistically significant 3% decrease in PE injuries (per person-year) following RM control interventions. While statistically non-significant, this 3% reduction represents approximately 18 fewer injuries. The RM process identified that while on-shift exercise was necessary for maintaining fitness, it lacked the necessary structure, guidance and management to ensure safe performance. Two retrospective cohort analyses on the current study’s population (Poplin et al., 2014; Poplin et al., 2016) found that over a five year span, “fit” and “less fit” commissioned FD employees were found to have an increased likelihood for injury than their “most fit” peers. However, PE, if performed improperly, frequently results in injury. As part of the PE RM control, there was increased emphasis placed on the responsibility of supervisors to assure everyone is fit for duty and can continue to perform all job functions without risking their own safety or that of their peers. The increased role of PFTs was also felt to be an effective component of reducing injuries and adding structure to exercise routines.

In our intervention FD, medical-related calls are the most prominent response activity, accounting for approximately 80% of responses and 16.9% of the reported injures (Poplin et al., 2012). It has been shown that firefighters providing EMS are at elevated risk of musculoskeletal injuries while performing PT (Lavender et al., 2000). Lifting, in particular, is associated with increased risk of back pain and is the most common cause of work-related low back injury (Chow et al., 2005). The implemented control strategies were focused on decreasing the potential for prolonged static postures and minimizing the repetitive loads and strains from lifting and moving patients in all ergonomic settings. It is not clear why these RM controls were not effective in reducing FD injuries. One key control strategy identified during the risk assessment, specifically new gurneys with electronic (vertical) lift assist technology, was not initially considered financially plausible. Near the conclusion of the study period, the FD was able to procure funds to outfit all ambulance apparatus with these gurneys; however, the timing of this purchase did not enable researchers to assess its effect in reducing injury or associated costs.

Before and throughout the timeframe of this study, the national rates of injury reported by the National Fire Protection Agency (NFPA) indicate that FG injuries continue to be the most prevalent activity associated with injury at approximately 46% of injuries (Karter and Molis, 2010; Karter and Molis, 2014). Coming into the study, the FD had demonstrated relatively low FG injury incidence, with only 11% of all injuries attributed to fire responses. Overall reported injuries in the U.S. fire service have been declining by approximately 2–5% per year since 2006, most likely due to fewer fires; however, the rate of injuries per FG response has not improved. For non-fire emergencies, despite an overall increase in the number of injuries reported since 1981, the rate of injury (per 1000 non-fire incidents) has declined (Karter and Molis, 2014). For the current study, risk assessments directed much of the attention of the control strategies toward post-suppression situational awareness and supporting peer safety checks. The fact that these strategies were identified by the participants of this research (and workforce employees) is indicative of their importance. The reduction in rate of FG injuries (approximately 9%) following with the RM program, although not statistically significant, suggests that the chosen interventions were efficacious or that other factors such as a potential Hawthorne effect may have contributed. The significant difference in FG injuries in the post-intervention period comparing across fire departments was likely a result of both decreasing injuries in the intervention and increasing injuries in the control department, although neither of these changes was statistically significant by themselves.

The current study had a number of limitations. The post-intervention time period was relatively short, and the individual control measures were introduced over the intervention time period rather than simultaneously. This lag-effect decreased evaluation time and thus statistical power to detect significantly significant changes in injury rates for the individual activities (PE, PT and FG) as well as overall injuries. As reflected in our original international research evaluating RM in coal mining (Poplin et al., 2008), it took multiple years for RM to achieve its full effectiveness. With fewer post-intervention years to evaluate costs, annual variation could contribute to a greater extent to the observed reductions in total, medical and indemnity costs. The inability to follow-up on the potential benefits of the lift-assist gurneys for PT serves as an example of the study’s limited time frame, as it is expected that continued gains would be observed if this RM effort were to be sustained and evaluated on a systematic, long-term scale. Furthermore, due to the recession, only limited internal FD funding was available, reducing the number and extent of controls implemented. While temporal trends in U.S. fire service injuries may have contributed to some of the changes observed in our intervention FD, overall injuries in our regional control increased over time, albeit non-statistically significantly. Finally, the RM program was limited in scope to specific firefighter duties, rather than the broad focus on all hazardous activities as conducted in the U.K. fire service. Based on our study findings and experience, RM should be sustained with dedicated resources and personnel assigned to the management and facilitation of the process. While this study was the first to prospectively evaluate RM within the fire service, future studies with greater resources and broader in scope are needed to validate and expand the reported findings.

## Conclusion

The results demonstrated favorable improvements in firefighter safety, workers’ compensation injury claims and costs, and complement what we have seen in other countries where similar RM methodologies have been institutionalized for all fire services. RM is a safety systems approach that, if adopted, should be initiated and designated a top priority by department administration and labor, and driven by an engaged workforce for effective solutions to be realized. However, the results of the current study suggest that even when limited in scope, RM has the potential to yield meaningful safety and health benefits in the U.S. fire service.

## Abbreviations

CPR: Cardiopulmonary resuscitation
EMS: Emergency medical services
FD: Tucson Fire Department
FG: Fireground
IAFC: International Association of Fire Chiefs
IAFF: International Association of Fire Fighters
PE: Physical exercise
PFT: Peer fitness trainers
PHLAME: Promoting healthy living: assessing more effects
POWR: Physician organized wellness regime
PPE: Personal protective equipment
PT: Patient transport
RM: Risk management
SOP: Standard operating procedure
WFI: Wellness-Fitness Initiative

## References

[CR1] Burgess JL, Duncan M, Mallett J, La Fleur B, Littau SR, Shiwaku K (2014). International comparison of fire department injuries. Fire Technol.

[CR2] Burgess JL, Duncan MD, Hu CC, Littau SR, Caseman D, Kurzius-Spencer M, Davis-Gorman G, McDonagh PF (2012). Acute cardiovascular effects of firefighting and active cooling during rehabilitation. J Occup Environ Med.

[CR3] Chow DH, Cheng IY, Holmes AD, Evans JH. Muscular and Centre of pressure response to sudden release of load in symmetric and asymmetric stoop lifting tasks. Appl Ergon. 2005;36(1):13–24. 10.1016/j.apergo.2004.10.001 15627417

[CR4] Gold MR, Siegel JE, Russell LB, Weinstein MC (1996). Cost-effectiveness in health and medicine.

[CR5] Griffin SC, Regan TL, Harber P, Lutz EA, Hu CC, Peate WF, Burgess JL (2016). Evaluation of a fitness intervention for new firefighters: injury reduction and economic benefits. Inj Prev.

[CR6] International Association of Fire Fighters (IAFF), International Association of Fire Chiefs (IAFC) (2008). The fire service joint labor management wellness-fitness initiative.

[CR7] Karter M, Molis J (2010). U.S. firefighter injuries – 2009.

[CR8] Karter M, Molis J (2014). U.S. firefighter injuries – 2013.

[CR9] Kuehl KS, Elliot DL, Goldberg L, Moe EL, Perrier E, Smith J (2013). Economic benefit of the PHLAME wellness programme on firefighter injury. Occupational Medicine-Oxford.

[CR10] Lavender S, Conrad K, Reichelt P, Johnson P, Meyer F (2000). Biomechanical analyses of paramedics simulating frequently performed strenuous work tasks. Appl Ergon.

[CR11] Leffer M, Grizzell T (2010). Implementation of a physician-organized wellness regime (POWR) enforcing the 2007 NFPA standard 1582: injury rate reduction and associated cost savings. J Occup Environ Med.

[CR12] Liboschik T, Fokianos K, Fried R (2017). Tscount: an R package for analysis of count time series following generalized linear models. J Stat Softw.

[CR13] Pollack KL, Poplin GS, Griffin S, Peate W, Day-Nash V, Nied E, Gulotta J, Burgess JL (2017). Implementing risk management to reduce injuries in the U.S. fire service. J Saf Res.

[CR14] Poplin G, Harris R, Pollack K (2012). Beyond the Fireground: injuries in the fire service. Inj Prev.

[CR15] Poplin GS, Miller HB, Ranger-Moore J, Bofinger CM, Kurzius-Spencer M, Harris RB, Burgess JL (2008). International evaluation of injury rates in coal mining: a comparison of risk and compliance-based regulatory approaches. Saf Sci.

[CR16] Poplin GS, Pollack KM, Griffin S (2015). Establishing a proactive safety and health risk management system in the fire service. BMC Public Health.

[CR17] Poplin GS, Roe DJ, Burgess JL, Peate W, Harris RB (2016). Fire fit: assessing comprehensive fitness in the fire service. Int Arch Occup Environ Health.

[CR18] Poplin GS, Roe DJ, Peate W, Harris RB, Burgess JL (2014). The Association of Aerobic Fitness with injuries in the fire service. Am J Epidemiol.

[CR19] TriData Corporation. The economic consequences of firefighter injuries and their prevention. Prepared for the National Institute of Standards and Technology and US Department of Commerce, NIST Report Number 05-874. 2005. https://www.nist.gov/publications/economic-consequences-firefighter-injuries-and-their-prevention-final-report.

[CR20] U.S. Department of Homeland Security, Federal Emergency Management Agency, U.S. Fire Administration National Fire Data Center. Firefighter Fatalities in the United States in 2014. August, 2015.

[CR21] US Department of Homeland Security, U.S. Fire Administration National Fire Data Center (2014). Fire-related firefighter injuries reported to the National Fire Incident Reporting System (2010-2012). Topical Fire Report Series.

[CR22] US Department of Labor, Bureau of Labor Statistics. Consumer price index. 2014. Available at: https://www.bls.gov/cpi/. Accessed 28 Feb 2014.

[CR23] Weiler MR, Lavender SA, Crawford JM, Reichelt PA, Conrad KM, Browne MW (2013). A structural equation modelling approach to predicting adoption of a patient-handling intervention developed for EMS providers. Ergonomics.

